# Effect of a high fat, high sucrose diet on the promotion of non-alcoholic fatty liver disease in male rats: the ameliorative role of three natural compounds

**DOI:** 10.1186/s12944-015-0087-1

**Published:** 2015-07-31

**Authors:** Sohair M. M. Ragab, Sary Kh. Abd Elghaffar, Tarek H. El-Metwally, Gamal Badr, Mohamed H. Mahmoud, Hossam M. Omar

**Affiliations:** Department of Zoology, Faculty of Science, Assiut University, Assiut, 71516 Egypt; Department of Pathology, Faculty of Veterinary Medicine, Assiut University, Assiut, Egypt; Department of Medical Biochemistry, Faculty of Medicine, Assiut University, Assiut, Egypt; Deanship of Scientific Research, King Saud University, Riyadh, Saudi Arabia; Food Science and Nutrition Department, National Research Center, Dokki, Cairo, Egypt

**Keywords:** Berberine, High-fat high-sucrose diet, Peroxisome proliferator-activated receptor γ, Non-alcoholic fatty liver disease, Dyslipidemia, O-coumaric acid, Quercetin

## Abstract

**Background:**

Non-alcoholic fatty liver disease (NAFLD) is a multifactorial disease with a complex pathophysiology. The clinical features of NAFLD include obesity, insulin resistance (IR) and dyslipidemia. Consumption of a diet high in saturated fats and sucrose is an important factor in the increasing occurrence of these metabolic disorders, primarily NAFLD and IR. We sought to assess the role of a high-fat, high-sucrose (HFS) diet in the promotion of NAFLD and to evaluate the effects of quercetin (Q), berberine (BB) and o-coumaric acid (CA) on modulation of these disorders.

**Methods:**

Fifty male rats were divided into 2 main groups as follows: group 1 comprised 10 rats fed a standard diet (SD), and group 2 comprised 40 rats fed an HFS diet for 6 weeks and then subdivided equally into 4 groups; one of these groups served as the HFS diet and each of the other three groups received daily supplementation with either Q, CA or BB for 6 weeks.

**Results:**

In the present study, several metabolic disorders were induced in our laboratory animal model, as evidenced by histological and biochemical changes. These alterations included serum and hepatic dyslipidemia (i.e., increased triglyceride, total cholesterol and low-density lipoprotein levels and decreased high-density lipoprotein levels), alterations in metabolic enzyme activities (lipase, glycerol-3-phosphate dehydrogenase, and glucose-6-phosphate dehydrogenase), histological changes in the liver (micro- and macrovesicular steatosis) and the downregulation of peroxisome proliferator-activated receptor γ (PPARγ) in adipose tissue and the liver. Daily oral supplementation with Q, CA or BB for 6 weeks after NAFLD induction had a hypolipidemic action and modulated metabolic markers.

**Conclusion:**

We showed that an HFS diet is able to promote NAFLD, and our results suggest that CA and BB are promising complementary supplements that can ameliorate the metabolic disorders associated with an HFS diet; however, Q requires further investigation.

## Background

High level of caloric intake has been associated with many diet-induced complications, including metabolic syndrome, cardiovascular disease and non-alcoholic fatty liver disease (NAFLD) [1]. Feeding a carbohydrate- and fat-rich dietary components have been used in rodents to induce the signs and symptoms of human metabolic syndrome [2]. NAFLD is a multifactorial disease with a complex pathophysiology. The clinical markers of NAFLD include obesity, insulin resistance (IR), and dyslipidemia [3]. Hepatic lipid dysregulation, oxidative stress, and pro-inflammatory cytokines interact synergistically to promote hepatic fat accumulation over time [4]. Dietary carbohydrates and fats of different nature, combinations and amounts have been used in various NAFLD induction studies [5–9]. Peroxisome proliferator-activated receptor γ (PPARγ) is a major metabolic transcription regulator particularly for hepatic lipogenesis [10]. The upregulation of hepatic PPARγ is frequently observed in mice fed a high-fat diet [11]. In addition, the liver-specific deletion of PPARγ in mice established the role of this transcription factor as a prosteatotic factor in the development of NAFLD [12]. Accordingly, PPARγ inactivation promotes the efflux of free fatty acids (FFAs) from the liver and muscle while increasing fat mass, which consequently increases insulin sensitivity [13].

Phytochemicals are bioactive compounds of plants that are not yet classified as essential nutrients despite their health-promoting properties [14]. The majority of edible phytochemicals possess cytoprotective antioxidative or anti-inflammatory activities [15]. Reportedly, their beneficial health effects also include; anti-obesity, lipid-lowering, and/or antidiabetic properties [16]. Phytochemicals include phenolic compounds, such as flavonoids (e.g., quercetin, epicatechin, rutin, myricetin, luteolin, naringenin, and silybin) [17]. As a mitochondrial antioxidant, quercetin has a wide range of biological effects, such as lowering blood pressure [18], reducing body weight [18], and ameliorating hyperglycemia-related diseases [19]. Phytochemicals also include phenolic acids present in plant-based foods such as fruits, vegetables, grains, tea, coffee, and spices and are consumed by most humans every day [20, 21]. Among these phenolic acids is coumaric acid (CA), which is a hydroxy derivative of cinnamic acid. CA exists in three isomeric forms, o-coumaric acid, m-coumaric acid, and p-coumaric acid [22]. The anti-adipogenic effect of o-coumaric acid appears to be mediated through the downregulation of the expression of adipogenic transcription factors (PPARγ and C/EBPα) and adipocyte-specific proteins (leptin), which suppresses dyslipidemia, hepatosteatosis and oxidative stress in obese rats [23]*.* Berberine is a botanical alkaloid found in the roots and barks of several plants, such as berberis, goldenseal (*Hydrastis canadensis*), and Coptis chinensis. It has been reported that berberine reduces body weight and significantly improves glucose tolerance and insulin action in obese and/or diabetic subjects [24, 25]. In addition to berberine enhancing insulin sensitivity, this compound reduces hyperlipidemia and ameliorates fatty liver [26]. The aim of this study was to investigate the ability of feeding rats with a high-fat, high-sucrose (HFS) diet to induce NAFLD and to assess the individual physiological roles of quercetin, berberine and o-coumaric acid in the modulation of NAFLD histological and biochemical progression.

## Materials and methods

### Animals

Fifty adult (six-week-old) Wistar rats (80-120 g) were purchased from the animal house of the Faculty of Medicine, Assiut University, Assiut, Egypt, and the animals were housed in cages in the animal house of the Zoology Department, Faculty of Science, Assiut University. All of the animal procedures were performed in accordance with the guidelines for the care and use of experimental animals established by the Committee for the Purpose of Control and Supervision of Experiments on Animals (CPCSEA) and the protocol of the National Institutes of Health (NIH). The animals were allowed to acclimate for 2 weeks before the experiment and were housed in metal cages in a well-ventilated room. The animals were maintained under standard laboratory conditions (25 °C, 60-70 % relative humidity and a 12-h light/dark cycle).

### Experimental design of diets and supplementation with natural compounds

After one week of acclimatization, the rats were randomly divided into 2 main groups: a control group of 10 rats that were fed a standard diet (SD; 80 % carbohydrates, 18 % proteins and 2 % fats) and 40 rats that were fed an HFS diet (55 % SD diet, 15 % beef tallow, 10 % sucrose, 5 % roasted peanuts, 5 % milk powder, 5 % egg, 3 % sesame oil and 2 % NaCl) plus 10 % sucrose in their drinking water. After six weeks of feeding, these 40 rats were subdivided into four groups. The first group (HFS) was left untreated; this was the positive control group. The other three groups were treated with quercetin (Q) (50 mg/kg b.w.), o-coumaric acid (CA) (75 mg/kg b.w) or berberine (BB) (50 mg/kg b.w). The quercetin and o-coumaric acid were dissolved in 20 % DMSO, whereas the berberine chloride was dissolved in a pre-warmed saline solution. These three compounds were purchased from Sigma-Aldrich, France and were orally administered daily for six weeks starting at six weeks of feeding with the HFS diet.

### Collection and preparation of samples

Animals were bled from jugular vein and serum was recovered after clotting by centrifuged at 6000 rpm for 1 h at 4 °C. Sera were aliquot stored at **-**80 °C. Rats in the different groups were killed by cervical dislocation. The liver was quickly removed. One part was fixed in 10 % neutral buffered formalin for the histopathological investigations. The other part was first frozen in liquid nitrogen and then stored at ^_^80 °C for later use in the biochemical studies. To prepare a 10 % w/v homogenate, 0.3 g of liver was homogenized in 3 ml of 0.1 M phosphate buffer (pH 7.4) using an IKA Yellow line DI homogenizer (18 Disperser, Germany). The homogenates were centrifuged at 6000 rpm for 1 h at 4 °C, and the supernatant were kept frozen at ^_^80 °C for subsequent biochemical assays.

### Serum lipid profile and hepatic lipids analyses

Serum triglycerides were determined enzymatically using commercially available reagent kits (Egyptian Company for Biotechnology (S.A.E), Cairo, Egypt) [27]. Total cholesterol was determined using a commercially available kit that was based on a modification of the cholesterol oxidase method [28]. High-density lipoprotein cholesterol (HDL-C) was determined using a commercial kit that was based on the precipitation method [29]. Low-density lipoprotein cholesterol (LDL-C) was calculated as total cholesterol – HDL-C-triglyceride x 0.2. Hepatic lipids were extracted according to the method of Folch et al. [30]*.* The total cholesterol (TCh) and triglyceride (TG) concentrations in the liver were analyzed with the same enzymatic kit that was used for the serum analysis.

### Activities of enzymes involved in lipid metabolism

The enzymatic activity of glucose-6-phosphate dehydrogenase (G6PDH) was measured in 2 ml of 50 mM Tris-HCl buffer (pH 7.4) containing 0.6 mM NADP, 2 mM glucose-6-phospate, 5 mM EDTA and the appropriate amount of sample at 25 °C. The reaction was monitored by measuring the change in absorbance at 340 nm. The enzymatic activity is expressed as units/mg protein. The activity of glycerol-3-phosphate dehydrogenase (G3PDH), a key lipogenic enzyme, was measured according to the method of Kozak and Jensen [31]. Briefly, the hepatic tissue was homogenized in 4 volumes of ice-cold 50 mM tri(hydroxymethyl)-aminomethane (Tris) buffer (pH 7.5) containing 1 mM EDTA, 1 mM β-mercaptoethanol and 0.5 % Triton X-100. After sequential centrifugations at 10,000 g for 15 min, the final supernatant fraction was collected as the source of enzyme. An appropriate amount of enzyme solution was incubated with 1 ml of substrate solution [100 mM triethanolamine-HCl buffer (pH 7.5) containing 2.5 mM EDTA, 0.12 mM NADH, 0.2 mM dihydroxyacetone phosphate and 0.1 mM β-mercaptoethanol], and the decrease in the absorbance at 340 nm was recorded over time; the enzymatic activity was expressed as units/mg protein.

Hepatic lipase activity was assayed spectrophotometrically using p-nitrophenyl palmitate (p-NPP) as substrate according to the method of Krieger et al. (1999) [32]**.** A quantity of 100 mg of hepatic tissue was homogenized in 1 ml of potassium phosphate buffer (pH 7.4) and then centrifuged at 8000 × g for 15 min; the supernatant was then collected as a sample. The substrate solution consisted of one part solution A (3.0 mM p-NPP in 2-propanol) and nine parts solution B [100 mM potassium phosphate buffer (pH 7) containing 0.4 % Triton-X100 and 0.1 % gum Arabic]. The reaction mixture (100 μl of sample + 1900 μl of substrate solution) was incubated at 37 °C for 20 min. The reaction was stopped by boiling for 10 min, followed by centrifugation at 8000 x g for 10 min. The release of p-nitrophenol was measured at 410 nm against a blank containing only buffer that was subjected to the same conditions. One unit of enzymatic activity is defined as the amount of enzyme that releases 1 μmole of p-nitrophenol per min under the conditions described above.

### Quantitative real time- PCR (QRT-PCR)

Total RNA was extracted from the liver and white adipose tissue using a QIA ampRNA blood Mini Kit (Cat. No. 52304, Gmbh) according to the manufacturer’s instructions. The RNA concentration and purity were assessed based on absorbance at 260 nm and 280 nm. QRT-PCR amplification was performed in a 96-well format with the Brilliant II SYBR Green QRT-PCR Master Mix Kit, 1-Step (Cat. No. #600825 single kit) using a real-time PCR system (Stratagene 3000P). The real-time RT-PCR primers were designed by the Primer Express 1.5 software (Invitrogen™), and the sequences were as follows: PPARγ, F, 5' -CAC AAG AGC TGA CCC AAT GGT TGC TG -3'; PPARγ, R, 5' -CGC AGA TCA GCA GAC TCT GGG TTC-3'. Quantification was performed by calculating the values of the Δcycle threshold (ΔCt) by normalizing the average Ct value of each treatment compared to its control and then calculating the 2^−ΔΔCt^ for each treatment.

### Histopathological examination and electron microscopic study

The 10 % neutral buffered formalin fixed tissue were routinely processed according to standard procedures. Then, sections (7 μm) of the different groups were mounted on slides and dried overnight at 37 °C. The sections were de-waxed in xylene, hydrated in a graded series of alcohol solutions and then stained with hematoxylin and eosin for histological evaluation. Small hepatic tissue fragments were cut into 1-mm^3^ sections. The fragments were immediately fixed in 2.5 % glutaraldehyde and rinsed in 0.1 M phosphate buffer. After fixation in 1 % osmium tetroxide and rinsing in 0.1 M phosphate buffer, the samples were dehydrated in a graded series of alcohol solutions and embedded in pure epoxy resin. Ultrathin sections (50-80 nm) were cut with a Leica AG ultramicrotome and stained with uranyl acetate and lead citrate [33]. The sections were examined with a TEM (Jeol, 100 CXII) operated at 80 KV at the Electron Microscopic Center, Assiut University.

### Statistical analysis

The data were tested for normality using the Anderson-Darling test and for homogeneity of variances prior to further statistical analyses. The data were normally distributed and were expressed as the mean ± standard error of the mean (SEM). Significant differences among groups were analyzed using a one-way ANOVA followed by a Newman-Keuls multiple comparisons test for multiple comparisons using PRISM statistical software (GraphPad Software). Differences were considered statistically significant at *p < 0.05*, 0.01, 0.001 or 0.0001.

## Results

### Effect of an HFS diet and treatments on biochemical measurements

**Fig. 1:**
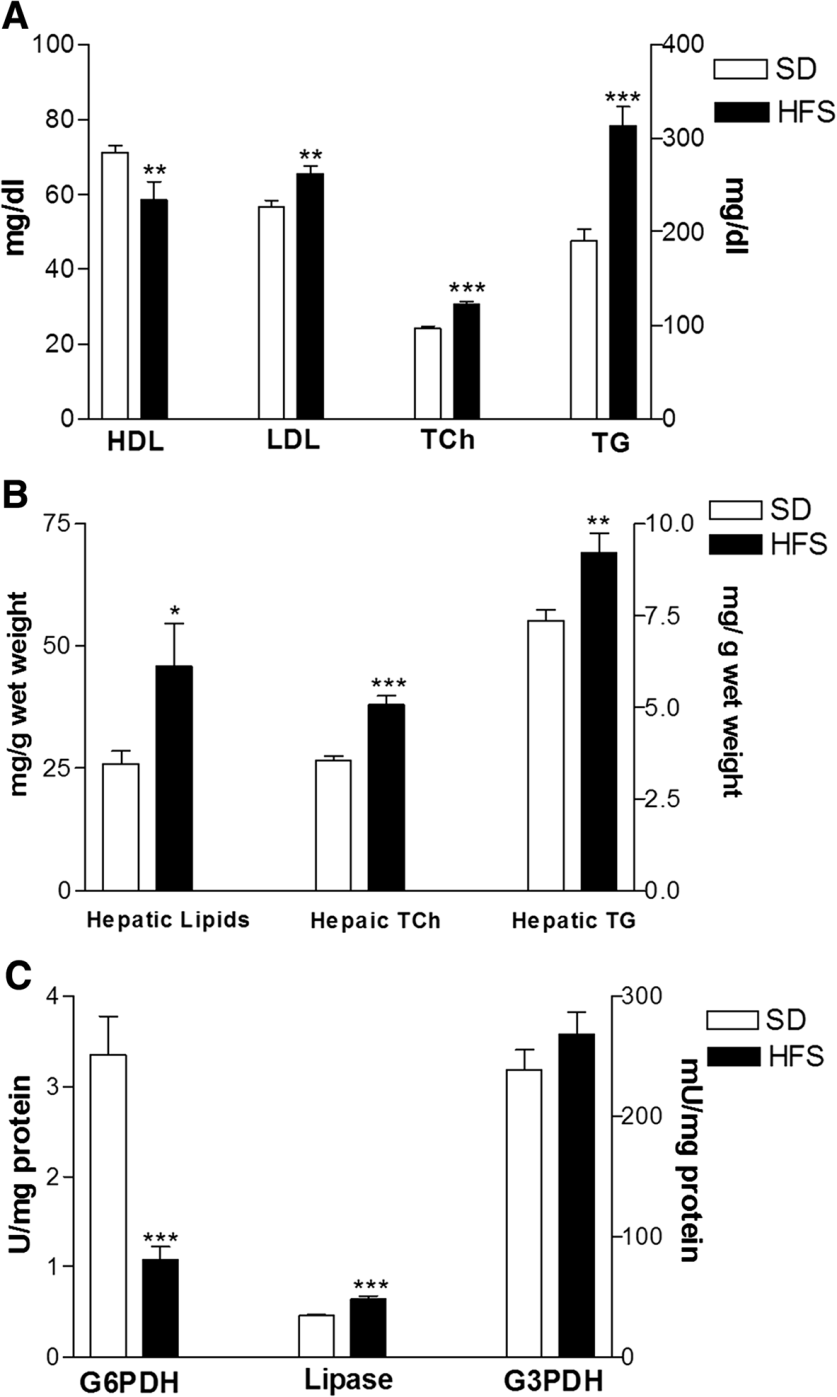
Effects of feeding rats an HFS diet on the serum lipid profile (**a**), hepatic lipids, TCh and TG (**b**), and hepatic metabolic enzymes (**c**) compared with those of SD-fed rats. The data are presented as the mean ± SE. * *p* < 0.05, ** *p* <0.01, *** *p* < 0.001. (ANOVA with Newman-Keuls multiple comparisons test)

**Fig. 2:**
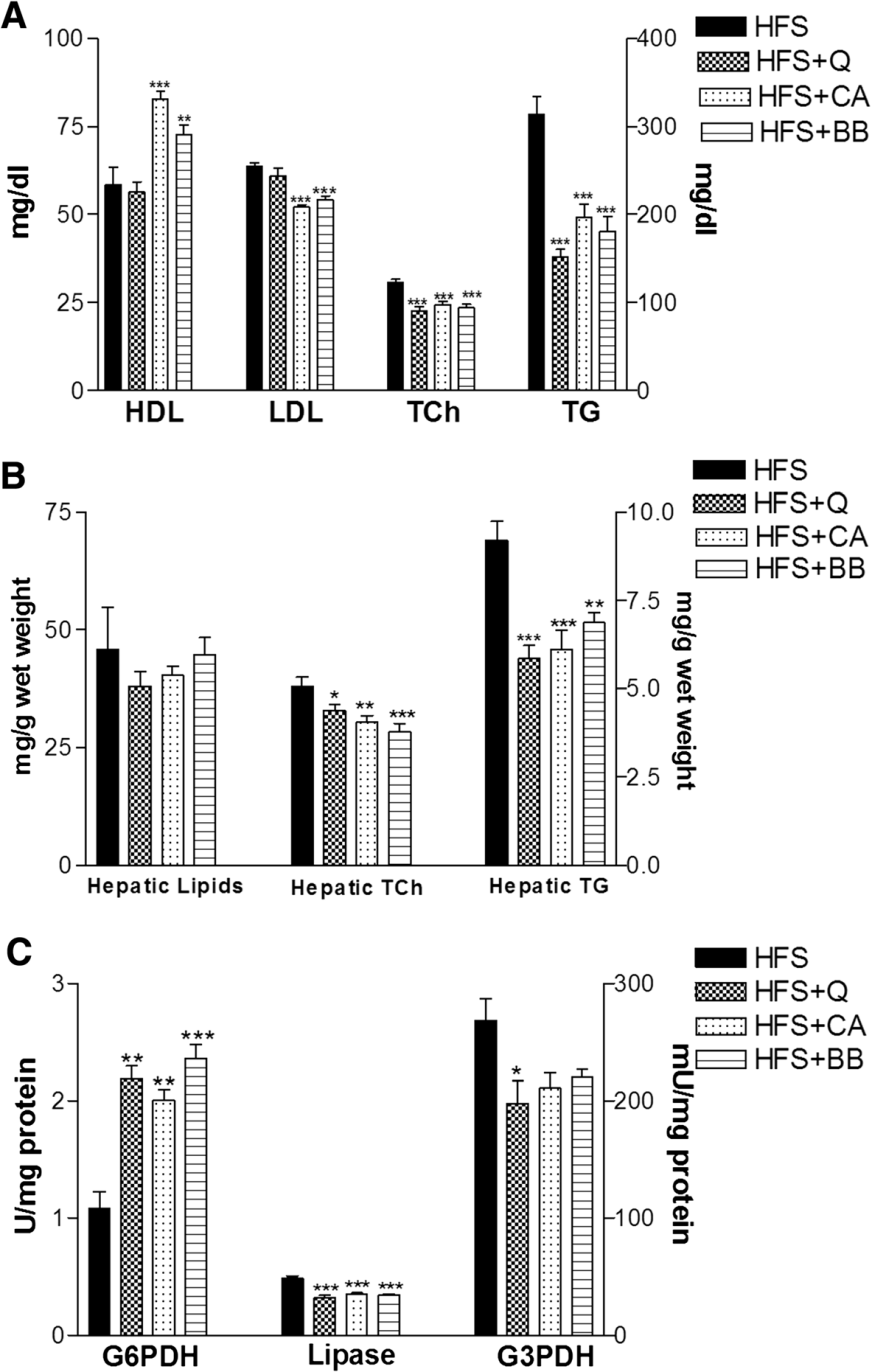
Ameliorative effects of Q, CA and BB on the serum lipid profile (**a**), hepatic lipids, TCh and TG (**b**), and hepatic metabolic enzymes (**c**) compared with the HFS-fed rats. The data are presented as the mean ± SE. * *p* < 0.05, ** *p* < 0.01, *** *p* < 0.001. (ANOVA with Newman-Keuls multiple comparisons test)

By the end of 12 weeks of feeding with the HFS diet, our data demonstrated that the pair-fed groups in the laboratory model exhibited differences in their serum and hepatic tissue lipid profiles. The HFS-diet-fed rats exhibited a significant increase in the levels of serum LDL (*p <* 0.01), TG and TC (*p <* 0.001) and a significant decrease in HDL (*p <* 0.01) compared with the SD-fed rats (Fig. 1a). Additionally, the HFS-fed rats displayed significant increases in hepatic lipids (*p* < 0.05) and TG (*p* < 0.01) and TCh (*p* < 0.001) content, as shown in Fig. 1b. Activity assays of the hepatic enzymes in the HFS-fed rats revealed a significant decrease (*p* < 0.001) in the G6PDH activity (U/mg protein), a significant increase (*p* < 0.001) in the lipase activity (mU/mg protein) and a non-significant difference in hepatic G3PDH (mU/mg protein) activity compared with the SD-fed rats (Fig. 1c). After six weeks of feeding rats the HFS diet, treatment with CA and BB resulted in significant decreases (*p* < 0.001) in the levels of serum LDL, TG and TC and a significant increase (*p* < 0.001) in the HDL level in the CA and (*p* < 0.01) BB groups, whereas treatment with Q significantly modulated (*p* < 0.001) the changes in serum TG and TC levels compared with the HFS-diet group, as shown in Fig. 2a. The hepatic lipid content was decreased in all of the treatment groups but not to a significant extent. Hepatic TCh content was significantly (*p* < 0.05) decreased in the Q group, the CA group (*p* < 0.01) and the BB group (*p* < 0.001). Additionally, the hepatic TG content was significantly (*p* < 0.001) lowered in the Q and CA groups, as well as in the BB group (*p* < 0.01), compared with the HFS group (Fig. 2b). The assays for hepatic enzyme activities revealed a significant increase in G6PDH activity (U/mg protein) in the Q and CA groups (*p* < 0.01) and in the BB group (*p* < 0.001). Hepatic lipase activity (mU/mg protein) was significantly decreased (*p* < 0.001) in all of the treatment groups, whereas G3PDH (mU/mg protein) activity was decreased significantly only in the Q group compared with the HFS-diet group (Fig. 2c).

### Effect of the HFS diet and treatments on histopathological observations

**Fig. 3:**
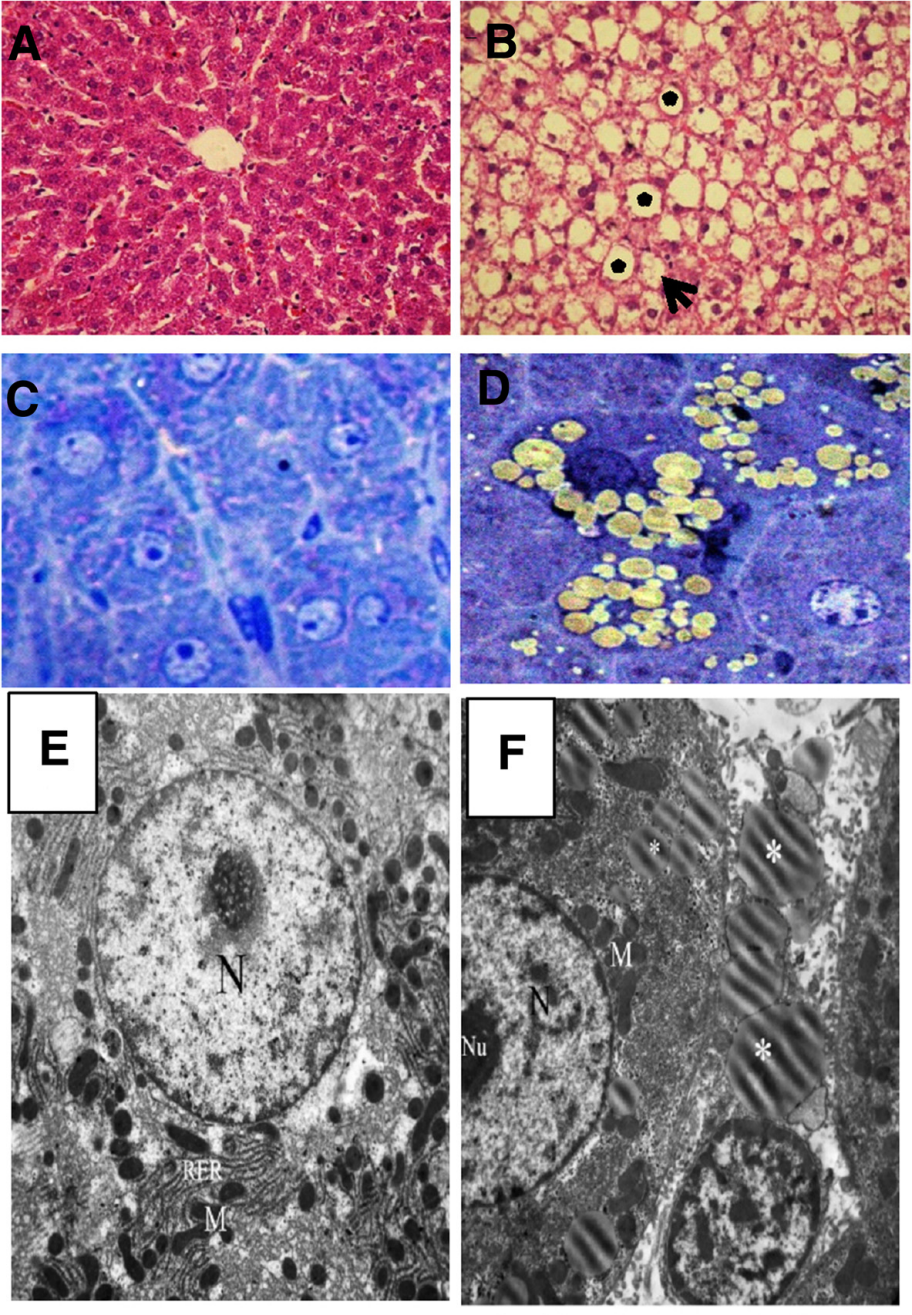
Histological and electron microscopy observations of the liver; sections stained with H&E: SD-fed rats (**a**) and HFS-fed rats with large (star) and small (arrow) lipid droplets (**b**). Semi-thin sections reveal microvesicular and macrovesicular steatosis in the liver of the HFS-fed rats (**d**) compared with the SD-fed rats (**c**). EM photos of clear and abundant lipid droplets in the HFS-fed rats (**f**) compared with the SD-fed rats (**e**) (5,800X)

The hematoxylin-and-eosin-stained sections revealed that the HFS diet led to an enlargement of the hepatocytes and an increase in the number of lipid droplets (Fig. 3b) in the liver compared to the SD group (Fig. 3a). In particular, the semi-thin sections exhibited microvesicular and macrovesicular steatosis of the liver of rats on the HFS diet (Fig. 3d) compared with the liver of an SD-fed rat (Fig. 3c). The electron microscopy study also revealed hepatocytes with abundant lipid droplets in the HFS-fed rats (Fig. 3f) compared with the livers of the SD-fed rats (Fig. 3e).

**Fig. 4:**
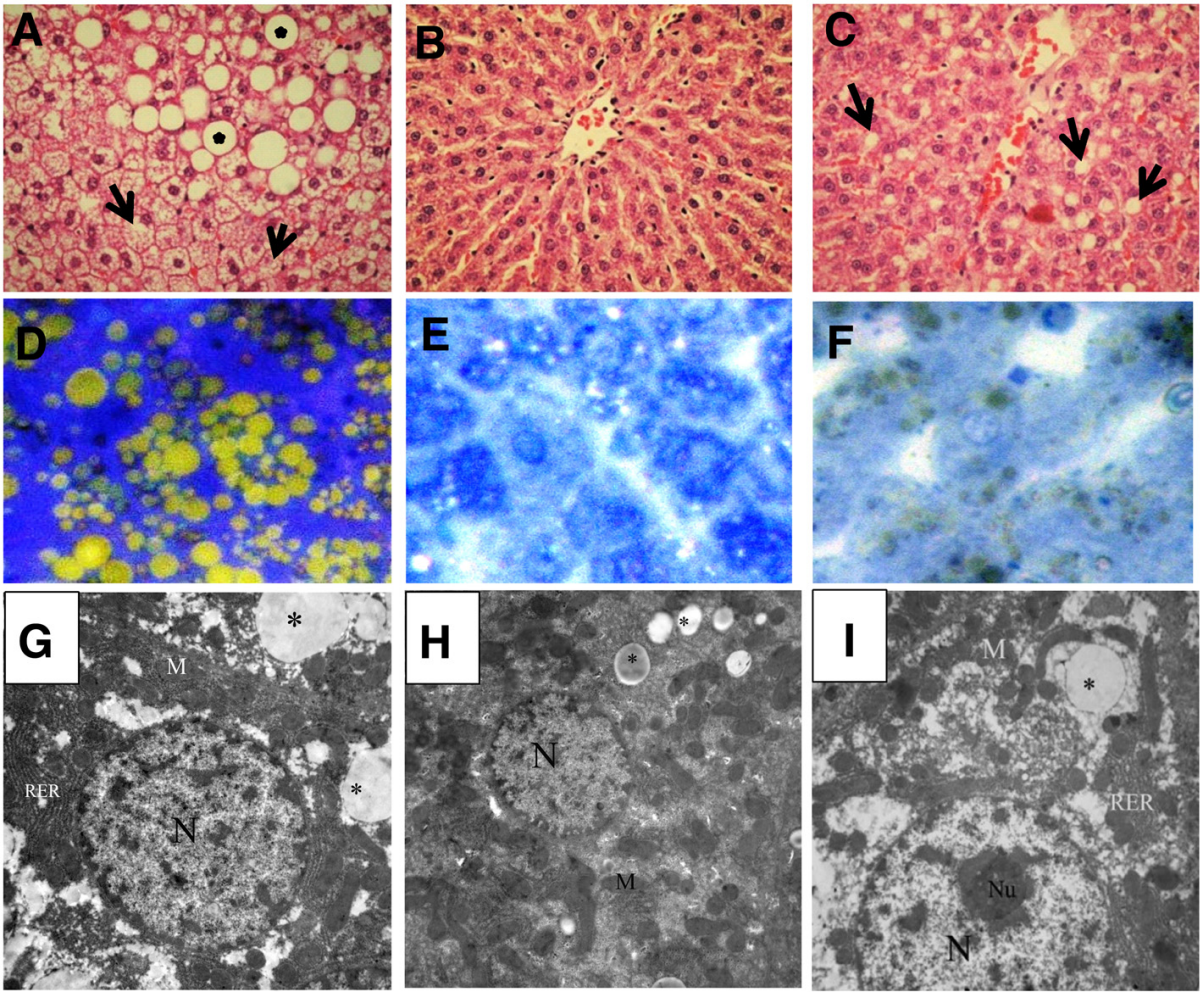
Histological and electron microscopy observations of the liver sections stained with H&E; the Q treated rats have microvesicular (star) and macrovesicular steatosis (arrow) (**a**); the CA group with fewer changes (**b**), and the BB group with small lipid droplets (**c**). The semi-thin sections reveal microvesicular and macrovesicular steatosis in the livers of the Q group (**d**), slight changes in the CA group (**e**) and microvesicular steatosis in the BB group (F). EM photos of clear lipid droplets with variable sizes in the Q, CA and BB groups (**g**, **h** and **i**), respectively

The quercetin-treated group exhibited macrovesicular steatosis with well-defined fat vacuoles (Fig. 4a); treatment with CA resulted in no marked changes (Fig. 4b) compared with the other treatments, whereas the BB group had fewer changes, represented by microvesicular steatosis, as shown in Fig. 4c. These observations were confirmed by the semi-thin sections, which revealed that treatment with Q did not ameliorate the effect of the HFS diet, as demonstrated by the abundant number of fat droplets (Fig. 4d), fewer steatotic changes than in the CA group (Fig. 4e) and the smaller fat droplets in the BB-treated group (Fig. 4f), compared with the HFS group (Fig. 2d). Additionally, the electron microscopy study revealed that the Q group had more fat droplets (Fig. 4g) than both the CA (Fig. 4h) and BB groups (Fig. 4i).

### An HFS diet modulates the expression of PPARγ

**Fig. 5:**
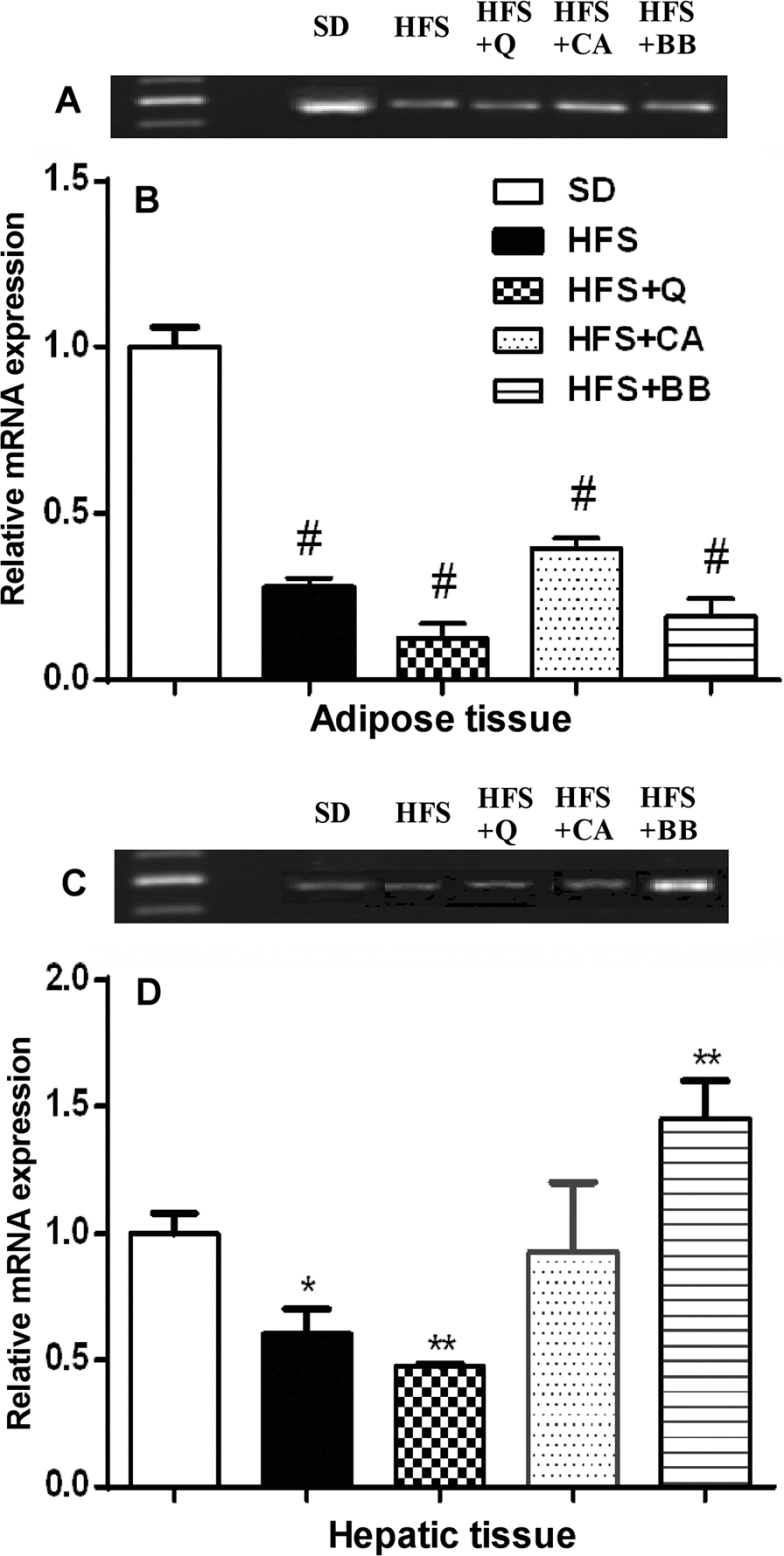
Effect of an HFS diet on the relative mRNA expression level of PPARγ in adipose and hepatic tissue, and the effect of the Q, CA and BB treatments on the modulation of these changes. The data are presented as one representative experiment (**a** and **c**) and accumulated data from three independent experiments are expressed as the mean ± SE (**b** and **d**). * *p* < 0.05, ** *p* < 0.01, *** *p* < 0.001, # *p* < 0.0001. (ANOVA with Newman-Keuls multiple comparisons test)

One representative PCR amplification products indicating the expression of PPARγ (Fig. 5a and c) are shown. This experiment shows that the expression of PPARγ in adipose tissues was obviously decreased in HFS-fed rats and the rats in all of the treatment groups compared with that in the SD-fed rats (Fig. 5a). However, in hepatic tissues of HFS- and Q-treated group the expression of PPARγ was clearly down-regulated as compared with SD-fed rats (Fig. 5c). In contrast, the expression of PPARγ in hepatic tissues was clearly up-regulated in the BB-treated group compared with the SD-fed rats. The rats of CA-treated group exhibit similar expression of PPARγ in their hepatic tissues. Accumulated data from three independent experiments (Fig. 5b and D) indicate that the HFS-fed rats and the rats in all of the treatment groups exhibited highly significant downregulation (*p* < 0.0001) of adipose tissue PPARγ expression compared with that in the SD-fed rats. PPARγ expression in hepatic tissues revealed significant downregulation in the HFS-fed rats and the Q group (*p* < 0.05, *p* < 0.01, respectively) and significant (*p* < 0.01) upregulation in the BB group compared with the SD-fed rats.

## Discussion

The model used in this study mimics third world diets, which mostly have a high carbohydrate and/or fat intake. The first observation in the present study was the non-significant change in the body weight of the SD-fed rats, HFS-fed rats and rats in all of the treatment groups. This result is in agreement with several studies that found that feeding with high-fat diets, high-sucrose diets or both did not increase the body weight of rats over the long term [34–36]. This can be explained by the prevalence of metabolically obese but normal-weight individuals who have obviously larger amounts of visceral adipose tissue associated with IR and other metabolic disorders [37]. In the present study, the HFS diet resulted in dyslipidemic changes as demonstrated by increased serum levels of TG, TCh and LDL and a lower level of HDL compared with the control rats; these findings may arise from the high fat content of beef tallow induced hypercholesterolemia [38]. We also checked the levels of serum insulin. We found that insulin level was increased in HFS, but was significantly reduced in Q-, BB- and CA- treated groups (data not shown). Dietary sucrose has been shown to significantly produce hypertriglyceridemia over the life spans of rats that had free access to food or were calorie restricted; this may be due to increased secretion of TG, which decreased the catabolism of the rats [39]. Treatment with Q, CA or BB modulated these alterations. Likewise, Teodoro et al. (2013) [40] proved the positive effect of BB supplementation (100 mg/kg/day) in the drinking water for 4 weeks on reversing the effects of feeding a high-fat diet for 12 weeks. Additionally, the daily oral administration of Q (50 mg/kg) for 4 weeks ameliorated the negative effects on the serum lipid profile of 4 weeks of feeding with 10 % fructose [41]. Previous studies have revealed that hydroxycinnamic acids (p-coumaric acid, caffeic acid, ferulic acid) and their derivatives efficiently improved hypercholesterolemia and type 2 diabetes [42]. With overnutrition and a lack of exercise, the liver and other tissues store excess energy as triacylglycerol (TAG). Shifting carbon energy into a storage form is likely protective against cytotoxic fatty acid (FA) accumulation. Peripheral IR may cause fatty liver by elevating the plasma levels of FA, glucose, and insulin, which stimulates hepatic lipid synthesis and impairs hepatic β-oxidation [43]. The present data revealed that the livers of the HFS-fed rats had significantly higher amounts of lipids than the livers of the SD-fed rats. In addition, measuring the hepatic TG and TCh lipid content revealed a highly significant increase in the HFS group compared with the SD group. These findings are in accordance with several previous studies that concluded that the multiple steps involved in lipid accumulation and inflammation in the liver occurred more rapidly in response to an HFS or HFD diet [5, 44, 45]. Although the present results indicated that treatment with Q, CA or BB failed to reverse the hyperlipidemia induced by the HFS diet, these compounds normalized the TG and TCh levels. Previous reports on this subject have suggested that saponins, such as diosgenin contained in fenugreek, form large micelles of bile acid and the saponin molecules in the small intestine, and these micelles inhibit cholesterol absorption by directly excreting cholesterol in the feces [46]. G6PDH plays a key role and is a crucial enzyme in the maintenance of the cellular redox potential and cell survival via the production of NADPH [47]. The data obtained from the present study clearly indicated that the HFS-fed rats exhibited a highly significant decrease in hepatic G6PD activity compared with the SD-fed rats. In addition, treatment with Q, CA or BB resulted in a significant recovery of the hepatic G6PD activity, importantly this recovery was significant in relation to the HFS group. In a recent study, plasma G6PD activity decreased with HFD-induced oxidative stress in rats. This decrease could be explained by hyperglycemia associated with the HFD, which caused the activation of protein kinase A and a subsequent increase in the phosphorylation and inhibition of G6PD activity and thereby a decrease in NADPH levels, leading to increased oxidative stress [48]. Hepatic lipase plays a major role in lipoprotein metabolism as a lipolytic enzyme that hydrolyzes TG and phospholipids in chylomicron remnants, intermediate-density lipoproteins (IDL), and HDL [49]. In the present study, hepatic lipase activity increased significantly in the HFS group compared with the SD group. Feeding with an HFS followed by co-treatment with an HFS and Q, CA, or BB significantly decreased hepatic lipase activity compared with the HFS group. These results are in agreement with those of Brunzell and Carr (2004) [50] who found that patients with the familial form of hypertriglyceridemia with central obesity usually have elevated hepatic lipase activity. The excessive accumulation of lipids within hepatocytes due to an imbalance between lipid formation and lipid degradation leads to hepatic steatosis [51]. Collectively, the present histopathological observations of the H&E-stained and semi-thin sections, confirmed by the electron microscopy study, verify the presence of microvesicular and macrovesicular steatosis of the livers of rats on the HFS diet compared with the livers of the SD-fed rats. Previous studies corroborate these observations; a fat- and sugar-enriched diet results in liver steatosis, lobular inflammation, hepatocyte ballooning, and portal inflammation [52]**.** Additionally, animal models fed on lard or beef tallow fats presented differences in the frequency of hepatic steatosis ranging from mild to severe based on the type of fats and feeding period [53, 54]. Recently, we explored the ameliorative effects of Q, BB and CA on the oxidative stress induced in NAFLD in rat model [55].

Treatment with Q revealed an ameliorative effect against the HFS diet as demonstrated by the abundant number of variable size fat droplets, fewer changes than in the CA group and the small fat droplets in the BB-treated group compared with the HFS group. The intragastric administration of BB at 187.5 mg/kg/day has been demonstrated to partially reverse the macrovesicular steatosis and inflammatory cell infiltration of portal areas and within hepatic lobules induced by a high-fat diet [54]. Previous studies have described the pivotal role of PPARγ in metabolism and glucose homeostasis [55–57]. In the present study, the results indicated that the HFS-fed rats and the rats in all of the treated groups exhibited highly significant downregulation of adipose tissue PPARγ expression compared with that in the SD-fed rats. However, PPARγ expression in the hepatic tissues revealed significant downregulation in the HFS-fed rats and the Q group and significant upregulation in the BB group compared with the SD-fed rats. The downregulation of PPARγ expression in the HFS-fed rats is in accord with several studies on different types of diets [58, 59]. Furthermore, PPAR-γ plays an essential part in lipogenesis of adipocytes by promoting the uptake of FFAs and increased the content of TAGs in the adipocytes and reduction of FFAs delivery to the liver [60]. Hepatic FFAs synthesis is catalyzed by acetyl-CoA carboxylase and fatty acid synthase, an enzyme that is complexly regulated by nuclear receptors (PPARα and PPARγ). Treatment with BB at 30 mg/kg has been found to increase the cardiac level of PPARγ mRNA expression in a rat model of hyperglycemia and hypercholesterolemia [59]. PPARγ expression has been shown to be remarkably decreased in response to 35 mg/kg streptozotocin and a 30-week feeding of a high-carbohydrate/high-fat diet; middle- and high-dose BB significantly returned the decreased PPARγ expression in diabetic adipose tissue to the control level [61]. Furthermore, certain natural PPARγ modulators, such as conjugated linoleic acid, can increase PPARγ expression, resulting in improved insulin resistance and glucose tolerance [62].

In conclusion, we have demonstrated that a high intake of fat and sucrose can induce NAFLD in male rats compared with the SD. Additionally, induction of NAFLD was exemplified by hyperlipidemia and metabolic disorders. Oral supplementation with natural compounds such as CA or BB ameliorated these disorders, but Q ameliorated only metabolic disorders In accordance with numerous previous studies supporting the benefits of various natural compounds in treating many diseases [63–70]. The present work provides experimental evidence to indicate that CA and BB can be considered promising complementary supplements for treating the development of hepatic steatosis associated with HFS diets.

## Abbreviations

BB: Berberine
CA: O-coumaric acid
FFAs: Free fatty acids
G6PDH: Glucose-6-phosphate dehydrogenase
G3PDH: Glycerol-3-phosphate dehydrogenase
HFS: High fat high sucrose
HDL-C: High density lipoprotein-cholesterol
IR: Insulin resistance
LDL-C: Low density lipoprotein-cholesterol
NAFLD: Non-alcoholic fatty liver disease
PPARγ: Peroxisome proliferator-activated receptor γ
Q: Quercetin
SD: Standard diet
TCh: Total cholesterol
TG: Triglycerides
